# Atypical functional connectome is associated with low reflective functioning in incarcerated adolescents

**DOI:** 10.3389/fpsyt.2024.1385782

**Published:** 2025-01-10

**Authors:** Mélodie Derome, Larisa Morosan, Patrick Heller, Martin Debbané

**Affiliations:** ^1^ Faculty of Psychology and Educational Sciences, University of Geneva, Geneva, Switzerland; ^2^ Translational Research Center, University Hospital of Psychiatry and Psychotherapy, University of Bern, Bern, Switzerland; ^3^ Division of Clinical Child and Adolescent Psychology, Institute of Psychology, University of Bern, Bern, Switzerland; ^4^ Division of Prison Health, Geneva University Hospitals, University of Geneva, Geneva, Switzerland; ^5^ Adult Psychiatry Division, Department of Mental Health and Psychiatry, Geneva University Hospitals, Geneva, Switzerland; ^6^ Research Department of Clinical, Educational, and Health Psychology, University College London, London, United Kingdom

**Keywords:** incarceration, RS-fMRI, reflective functioning, psychopathy, delinquency, externalizing behaviors

## Abstract

**Introduction:**

While functional neuroimaging studies have reported on the neural correlates of severe antisocial behaviors, such as delinquency, little is known about whole brain resting state functional connectivity (FC) of incarcerated adolescents (IA). The aim of the present study is to identify potential differences in resting state connectivity between a group of male IA, compared to community adolescents (CA). The second objective is to investigate the relations among FC and psychological factors associated with delinquent behaviors, namely psychopathic traits (callous unemotional traits, interpersonal problems, and impulsivity), socio-cognitive (empathy and reflective functioning RF) impairments and psychological problems (externalizing, internalizing, attention and thought problems).

**Methods:**

31 male IA and 30 male CA participated in 8 minutes resting state functional MRI. Network Based Statistics (NBS) was used to compare FC among 142 brain regions between the two groups. Correlation and regressions analysis were performed to explore the associations between FC and the self-reported psychopathic traits, empathy, RF, and psychological problems.

**Results:**

Compared to the CA, the IA group presented significantly increased resting state FC in a distributed subnetwork including medial prefrontal, posterior and dorsal cingulate, temporal, and occipital regions. Both within the IA group and across the whole sample, increased mean connectivity of the subnetwork correlated with lower RF (RF uncertainty). Across the whole sample, the mean connectivity was associated with higher scores of externalizing problems and impulsivity dimension of psychopathy.

**Discussion:**

While extending the characterization of whole brain resting state FC in IA, our results also provide insights into the neurofunctional mechanisms linking low reflective functioning abilities to externalizing behavior during adolescence.

## 1 Introduction

Youth delinquency represents a global public health challenge, leading to negative consequences for individual development (e.g. legal problems, health problems, low educational attainment, low socio-economic status), for family functioning, and for society in general (1). Although increased efforts have been invested to reduce delinquent behaviors in youth, current intervention and prevention strategies have proven to be limited in their efficiency (2). For this reason, a large body of research focused on the psychological factors underpinning youth delinquency. Delinquent behaviors have been consistently associated with high psychopathic traits, described by three main dimensions - emotional callousness (CU), impulsivity and interpersonal manipulative behaviors (3, 4). The impulsivity dimension of psychopathy has been linked to reactive forms of delinquency, probably underpinned by difficulties in emotion regulation and impulse control (5, 6). Furthermore, previous studies have suggested that individuals with high CU and interpersonal manipulative traits are associated with calculated, goal-oriented, proactive delinquent behaviors (7–9). Of note, impulsivity is not specific to delinquency but rather a shared trait in externalizing disorders [e.g. ADHD, conduct disorder, substance abuse (10)].

Youth delinquency has also been previously associated also with impairments in social-cognitive processes, such as reduced empathy, as in inability to feel and understand others’ emotions (11–13) and reduced reflective functioning (14, 15). Reflective functioning (RF) refers to the capacity to recognize that one’s own and others’ behaviors are motivated by internal states (e.g. emotions, thoughts, desires etc.) (16). Thus, reduced RF represents the difficulty to reflect on behaviors in terms of mental states, difficulties that can be assessed using the subscale of uncertainty about mental states of the Reflective Functioning Questionnaire (17). Furthermore, several epidemiological studies have indicated higher rates of psychological problems in adolescents presenting delinquent behaviors (18, 19), some of which (e.g. attention and hyperactivity problems and conduct problems) put youth at increased risk of committing delinquent acts (20). Although a large body of research has focused on the psychological factors, little is known about the neural correlates of youth delinquency. Investigating the neural processes and their correlates with psychological risk factors can provide an integrative understanding of the mechanisms sustaining delinquent behaviors in youth, which may stimulate further development of prevention and intervention strategies.

A promising approach to understand the neural correlates of behavioral and psychological processes is to conceptualize and study the brain as a network of interconnected regions (21), by using resting state functional magnetic resonance imaging (rs-fMRI). rs-fMRI measures the spontaneous fluctuations of blood oxygen level-dependent (BOLD) signals of various regions (22, 23) to assess the brain’s patterns of functional organization “at rest”, in the absence of any explicit task (24). Focusing on the resting state FC in delinquent, incarcerated adolescents (IA) compared to non-delinquent, community adolescents (CA) will help us understand the specific default functional connectivity (FC) associated with delinquent behaviors in youth, as well as less extreme maladaptive behaviors. Compared to the CA, IA previously presented both *increased* and *decreased* resting state FC between brain regions previously identified as part of different brain networks, implicated in self-referential thinking (e.g. default mode network DMN), impulse control (e.g. executive control network ECN) and integration of sensorial information (e.g. salience network- SN). Chen and colleagues showed dysfunction in a right medial prefrontal-caudate circuit (25), further Aghajani and colleagues found psychopathic trait-specific increases and decreases between the amygdala subnetwork and respectively cortical midline structures, and frontolimbic network (26, 27). Finally, Sun and colleagues observed default mode network alterations with increased FC in posterior cingulate and decreased FC in middle temporal, angular, precuneus and middle frontal cortex (28).

Furthermore, previous studies report that the differences in FC observed in IA could be partially explained by the presence of psychological and personality factors. For instance, IA presenting high scores on the emotional callousness dimension of psychopathy showed reduced FC between brain regions previously identified as part of DMN and SN (26, 27, 29). On the other hand, the presence of high impulsivity and interpersonal psychopathic traits have been associated with increased connectivity in executive and motor networks, but also in DMN and SN networks (30, 31). Although these studies provided some insights into the neural correlates of psychopathic traits in IA, including the lack of empathy present in CU traits, rs-FC associations with other psychological factors associated with delinquent behaviors in youth (e.g. RF) as well as with different psychological problems presented by IA have yet to be examined.

Understanding the neural correlates of socio-cognitive processes and psychological problems related to delinquent behaviors is especially important during adolescence, a critical period in the development of socio-cognitive processes (32) and for the onset of a wide range of psychological problems (33). Furthermore, most previous studies focused on *a priori* defined brain regions and networks, overlooking the FC of more distributed network of brain regions. Using a whole-brain approach widens our understanding about functional architecture in IA compared to a group of CA.

The present study aims to map the potential differences between incarcerated (IA) and community (CA) male adolescents in rs-FC among a comprehensive set of 142 distinct cortical and subcortical brain regions, without defining *a priori* brain networks. For this purpose, we used a whole-brain connectivity approach, namely the Network Based Statistics (NBS) (34, 35). NBS maps regional brain networks that show a significant between-group differences in FC, by identifying inter-regional brain connections that are associated with a contrast of interest (in this case, IA vs. CA). The idea behind NBS is to utilize the presence of connections associated with such contrast of interest to potentially yield greater power than is possible by correcting the p-values computed for each connection comprised in the graph. We expect to identify differences in FC within specific subnetworks (e.g. DMN, SN, ECN). Given the mixed results of previous studies regarding the nature of resting state FC differences (increased or decreased connectivity) between IA and CA, and that the present study is the first to use a whole-brain NBS approach to study resting state FC in delinquent adolescents, we do not hypothesize any specific directions of FC differences between the two groups. Resting-state functional MRI (rs-fMRI) studies have employed various analytical approaches to investigate neural connectivity in populations with behavioral and cognitive impairments. Seed-based analyses, which focus on pre-defined regions of interest, have been instrumental in identifying focal alterations in connectivity, particularly in regions like the amygdala and prefrontal cortex that are implicated in emotion regulation and impulse control. For instance, studies of delinquent juveniles with high callous-unemotional (CU) traits have reported reduced connectivity between the amygdala and prefrontal regions, aligning with deficits in emotional processing and moral reasoning (26). However, this method is constrained by its reliance on *a priori localized*, single-seed hypothesized pathways of connectivity, potentially overlooking broader network-level disruptions. Independent Component Analysis (ICA), another commonly used approach, enables the decomposition of functional networks, such as the default mode network (DMN) and salience network (SN). Studies using ICA have observed reduced DMN connectivity in adolescents with conduct disorder and externalizing behaviors, implicating deficits in self-referential processing (36). While powerful, ICA results can vary depending on methodological choices, such as the number of components extracted, and may not capture inter-network interactions.

In contrast, the Network-Based Statistic (NBS) method used in this study addresses these limitations by identifying significant subnetworks of connectivity differences without the need for predefining regions or networks. This data-driven approach is particularly suited to capturing distributed connectivity patterns across the brain, which may reflect the heterogeneity of delinquent behaviors and their neurobiological correlates. For example, adolescents with externalizing behaviors often exhibit impairments across multiple functional networks, including the DMN, SN, and executive control networks (ECN), suggesting that a whole-brain approach like NBS is critical for detecting these complex and interconnected patterns.

A secondary aim of the present study is to investigate the associations among resting state FC and psychopathic dimensions (CU, impulsivity, and interpersonal problems), socio-cognitive processes (empathy and reflective functioning), and different psychological problems (externalizing, internalizing, attention, and hyperactivity and thought problems). We expect distinct associations among psychopathy dimensions and resting state connectivity. Based on previous evidence, we hypothesized that the callousness dimension would be associated with reduced resting state FC, whereas the impulsivity and interpersonal dimensions would correlate with increased FC. This hypothesis stems from previous literature on the neurobiological underpinnings of psychopathy, where callous-unemotional traits have been linked to reduced connectivity in brain networks related to emotional regulation (e.g. DMN), suggesting a deficit in affective processing. In contrast, impulsivity, a common feature across externalizing disorders (e.g. ADHD, conduct disorder), has been associated with hyperconnectivity in areas implicated in reward processing and executive function (37, 38). Given the novelty of our study, investigating the relations among resting state connectivity and socio-cognitive processes and psychological problems, we do not hypothesize any specific direction of these associations.

## 2 Methods and materials

### 2.1 Participants and procedure

Thirty-one incarcerated male adolescents (IA; *M*age=16.05, *SD*=1.17) placed in the observation sector of a juvenile detention in Geneva, Switzerland and thirty community male adolescents (CA; *M*age=16.11, *SD*=1.19), matched for age, took part in the study. The IA were placed in the juvenile detention center after they received a decision from the juvenile court, depending on their offence severity and delinquent history. Adolescents were in detention, meaning they were not allowed to exit the center without the permission of a judge. They were lodged into locked, separate rooms in the center and they were under constant supervision from the custody agent and educators from the center. Throughout the day, the detention center offered educational and professional guidance. Adolescents had to attend compulsory school classes taking place in the center or vocational classes (e.g. woodwork, cooking) inside or outside of the detention center.

The offense record from IA included different types of offenses: runaways and misconduct (51.6%), drug offenses (48.4%), driving offenses (12.9%), thefts (45.2%), property destructions (22.6%), robbery (12.9%), physical aggression (51.6%). The majority (91.3%) of IA had committed more than one offense, with an average number of offences at 2.5 (between 1 and 5 offenses). Seventeen IA committed at least one violent offence (robbery or/and physical aggression), whereas 14 adolescents committed only non-violent offenses. IA who committed at least one violent offense did not differ significantly from the IA without violent offenses on any variable of interest in the study (p>0.209). The sample partially overlaps with those reported in our previous behavioral studies (14, 39).

The IA were recruited by the juvenile detention center staff under the supervision of P.H. and they were individually tested at the center facility in a private room, by trained psychologists and research assistants, under the supervision of M.D. and L.M. The CA were recruited via advertising leaflets and were tested individually at our research unit. The exclusion criteria were intellectual disability and psychotic disorders. Both groups completed the self-report questionnaires and the cognitive functioning evaluation, before the neuroimaging part of the study, which was performed at the University Hospital of Geneva. The IA completed the MRI testing at an average interval of 64.84 days (*SD*= 21.97) since their arrival in the detention center. Written informed consent was obtained from all the participants and, for participants under 18 years old, also from their legal guardians. All adolescents participated voluntarily and received monetary compensation (50 CHF) at the end of their assessment. The protocol was approved by the Institutional Review Board of the Department of Psychiatry of the University of Geneva Medical School.

### 2.2 Instruments assessing psychological characteristics

*Psychopathic traits* were assessed using Youth Psychopathic Traits Inventory (YPI; 40). The 50-item questionnaire evaluates three dimensions of psychopathy, each consisting of several subscales: affective dimension assessing callous-unemotional traits (e.g. “To be nervous and worried is a sign of weakness”), impulsivity dimension (e.g. “It often happens that I do things without thinking ahead”) and interpersonal dimension, assessing grandiose, manipulative behaviors (e.g. « It is easy for me to manipulate other people »). The French translation of YPI presented a Cronbach alpha between 0.73 and 0.9.

*Empathy* was measured using the Basic Empathy Scale (BES; 41). BES assesses cognitive empathy- the ability to understand emotional states in others (e.g., “I easily realize when my friends are scared”) and affective empathy- the ability to share others’ emotional states (e.g., “When I am with a friend who is scared, I become scared”). The BES was validated in French, presenting a Cronbach’s alpha between 0.66 and 0.8 (41).

*Psychological problems* presented in the last 6 months were assessed using the French version of Youth Self-Report (YSR; 42). We selected the main categories of psychological problems measured by the YSR: externseedalizing behaviors (aggressive behaviors and rule-breaking behaviors), internalizing problems (withdrawal, anxiety, depression, and somatic complaints), attention deficit and hyperactivity problems (ADHD), and thought problems (bizarre behaviors and thoughts, anomalous perceptions). The YSR is validated in French, presenting good internal consistency with a Cronbach’s alpha ranging between 0.83-0.92 (43, 44).

*Reflective Functioning* was measured using the French version of the Reflective Functioning Questionnaire (RFQ; 17). The RFQ measures two subscales, the certainty (RFQc) and the uncertainty (RFQu) about mental states. Based on recent evidence that the RFQ might assess a unidimensional construct (e.g. uncertainty about mental states) and that a double-scoring of the same items is not recommended (45–47), in the present study we used only the RFQu subscale. RFQu indicates the extent to which the participant agreed with statements such as “I don’t always know why I do what I do”, high scores indicating a lack of use of mental states in order to explain behaviors. High score of RFQu represent a lack of knowledge about mental states and impairments in linking mental states to behaviors (48). The Cronbach’s alpha for the RFQu subscales of the French version of RFQ validated in adolescent samples was 0.67 (17).

### 2.3 Covariates

*Verbal abilities* were assessed using the French version Vocabulary subtest of the Wechsler Intelligence Scale for Children (WISC) Fourth edition (49). The Vocabulary subtest measures word knowledge, language development, and concept understanding and it was selected as a covariate in the present paper based on previous studies suggesting that low verbal abilities are strongly associated with delinquent behaviors (50).

The *number of offences* that the IA were convicted for was calculated based on the official legal records of the juvenile detention center. *Time since incarceration* was calculated for each participant as the time difference between the neuroimaging testing date and the date of the admission in the center.

### 2.4 MRI data acquisition and analysis

#### 2.4.1 *Image a*cquisition

MRI images were acquired on a 3T Siemens Scanner. The T1-weighted sequence was collected with a 3D volumetric dimension using the following parameters: TR = 2500 ms, TE = 3 ms, flip angle = 8 degrees, acquisition matrix = 256 x 256, field of view = 22 cm, slice thickness = 1.1 mm, 192 slices. For the resting state fMRI sequence, subjects were asked to keep their eyes open, fixate a cross on the screen, let their thoughts wander and refrain from falling asleep for the duration of the 8-minute scan. Head movement was minimized during scanning with a comfortable vacuum cushion constraint. The 200 blood-oxygenation-level-dependent (BOLD) images were acquired as follow: TR = 2400 ms, TE =30 ms, 38 axial slices, slice thickness = 3.2 mm, flip angle = 85°, acquisition matrix = 94 x 128, field of view = 96 x 128.

#### 2.4.2 Preprocessing

The pre-processing of rs-fMRI images was conducted using the Data Processing Assistant for Resting state fMRI (DPARSF) which is based on Statistical Parametric Mapping (SPM12) and Resting-state fMRI data Analysis Toolkit (REST). The first 10 images in each participant were discarded to remove T1 equilibration effects, and the remaining 190 images were corrected for slice timing and realigned to the mean image to correct for motion artifacts. All participants showed less than 2mm of displacement on average and 2° in their 6 head motion parameters. In addition, frame wise displacement (Power) did not differ between the 2 groups (t(45)= -1.616, p=0.113). T1-weighted anatomical images of each subject were co-registered to the mean realigned functional images and segmented in 6 outputs (gray and white matter, CSF, bones, skin and air). The registration step employed a Diffeomorphic Anatomical Registration using Exponential Lie algebra (DARTEL) to create a population-specific template. Following normalization, spatial smoothing was applied using an isotropic Gaussian smoothing kernel with a full width at half maximum (FWHM) of 6mm in order to decrease spatial noise prior to statistical analyses. The data were then detrended and low frequency fluctuations (0.01-0.08 Hz) were filtered to reduce the effect of noise. Six head motion parameters, white matter signal and cerebrospinal fluid signals were regressed from the filtered BOLD signal.

#### 2.4.3 Network construction

The data were extracted from 160 brain regions [based on the Dosenbach’s functional atlas (51)]. The cerebrum of pre-processed fMRI scans was parcellated according to a segmentation method in which each region was functionally independent. Because of incomplete coverage of the cerebellum in the dataset, only cerebral regions were parceled (cortical and subcortical), with 142 regions of interest (ROIs). Pair-wise associations were calculated resulting in a 142x142 connectivity matrix for each participant. Pearson correlation coefficient was used to quantify each pair-wise association. The Pearson’s r values were normalized to Z scores using Fisher’s transformation, each cell of the correlation matrix representing the strength of the connection (edge) between two nodes.

#### 2.4.4 Network analysis NBS

The network-based-statistic (NBS) was used to identify regional brain networks showing a significant between-group difference in inter-regional FC. This toolbox has been specifically designed to test hypotheses in the connectome and search for differences in inter-group connectivity. This method has been previously employed to investigate the neurobiological basis of other psychological and developmental disorders, such as borderline personality disorder (52) and autism spectrum disorders (53). Firstly, a t-test is performed to test for between-group differences in the correlation coefficient at each of the 142 x (142-1)/2 = 10011 unique regional pairings, this step specifies the hypothesis to be tested at every connection with the general linear model. For each of these associations, the t-test contrasting the two groups is calculated independently using the values stored in each subjects’ connectivity matrix. The connections that exceed this threshold are those susceptible of showing significant differences in FC between IA and controls. Interconnected networks, known as graph components, were identified among the connections with a t-statistic exceeding a threshold of 3.5. Most of the studies using NBS applied a threshold between 3 and 3.5, thus we decided to use a more conservative threshold, to identify the most robust differences. Family wise error (FWE) corrected p-value was calculated for the size of each resulting component using permutation testing (20 000 permutations). For each permutation, the group labels were randomly shuffled, and the largest interconnected network was identified. FWE-corrected p-value was estimated for each interconnected network as the proportion of permutations that yielded a larger interconnected network or one of equal size. We tested two alternative hypotheses independently: 1) CA>IA, 2) IA>CA.

### 2.5 Statistical analysis - psychological characteristics

**Figure 1:**
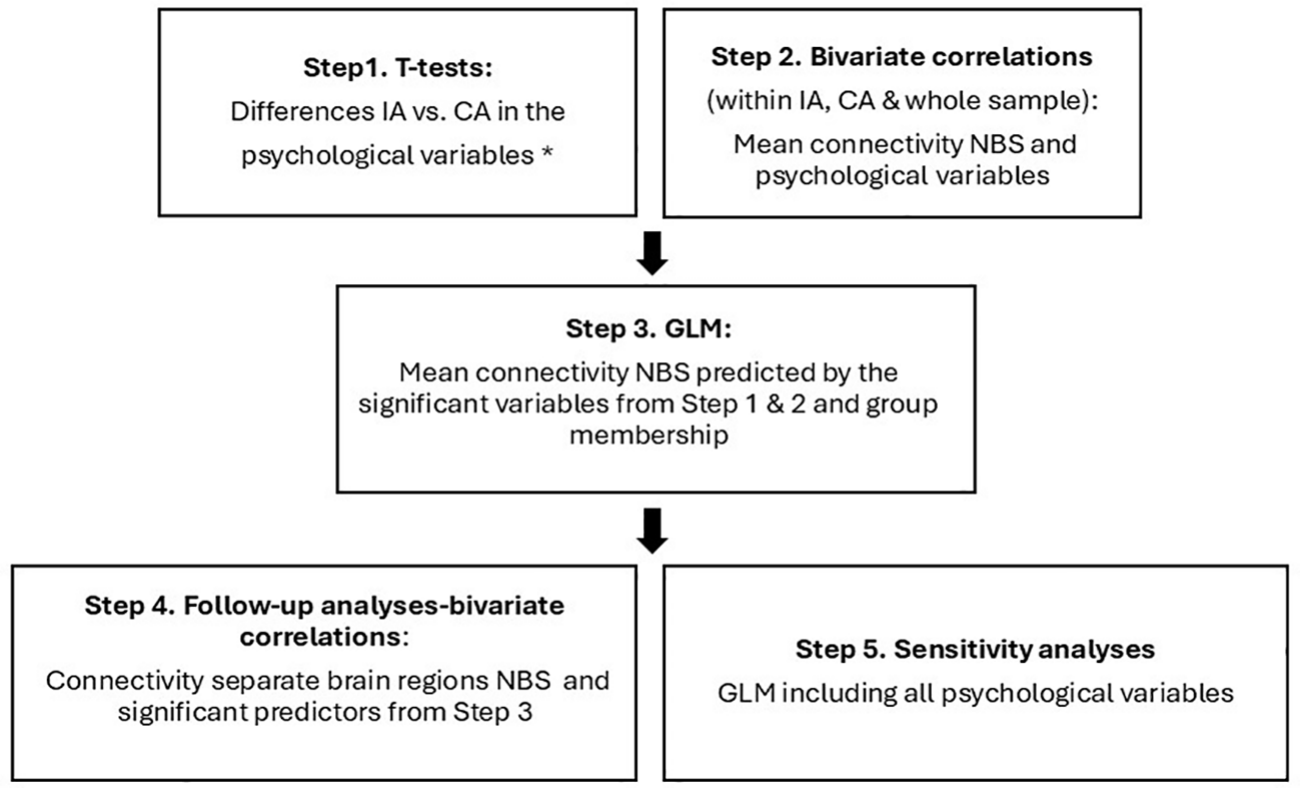
The steps of the statistical models used in the study to analyze the psychological variables. * WISC: Vocabulary; BES: affective, cognitive empathy; RFQ: Uncertainty about mental states; YSR: externalizing, internalizing, ADHD, thought problems; YPI: CU, interpersonal, impulsivity.

The statistical analysis for the psychological variables were performed in several steps, presented in **Figure 1**. T-tests were used to compare the two groups on the psychological characteristics (psychopathic traits, reflective functioning, empathy, psychological problems, and verbal abilities). Several participants had missing data in different measures, and they were removed case-wise in the analyses. **Table 1** presents the number of participants that completed each measure.

**Table 1:** Descriptive statistics and the results of t tests for behavioral variables, as well as the number of participants that completed each measure.

	Incarcerated adolescents		Community adolescents		*t* value	95% CI	*p*
	N	M (SD)	N	M (SD)			
Age	31	16.05 (1.17)	30	16.11 (1.19)	0.20	-0.54 to 0.66	0.841
WISC vocabulary	31	8.23 (3.29)	28	11.43 (3.29)	3.73	1.48 to 4.92	**<0.001**
YSR externalizing	31	69.97 (8.19)	29	56.45 (9.71)	-5.84	-18.15 to -8.88	**<0.001**
YSR internalizing	31	55.35 (11.11)	29	52.83 (10.37)	-0.90	-8.09 to 3.03	0.367
YSR ADHD	31	60.29 (7.86)	29	57.03 (6.89)	-1.17	-7.08 to 0.57	0.094
YSR thought problems	31	62.16 (7.57)	29	59.07 (9.47)	-1.40	-7.55 to 1.32	0.167
RFQu	30	4.13(2.80)	25	2.24 (2.65)	-2.55	-3.37 to -0.40	**0.013**
YPI CU	31	10.83 (2.43)	28	10.19 (1.62)	-1.18	-1.74 to 0.44	0.240
YPI impulsivity	31	14.81 (1.98)	28	12.07 (2.45)	-4.74	-3.92 to -1.58	**<0.001**
YPI interpersonal	31	9.69 (2.63)	28	10.05 (2.19)	0.56	-0.90 to 1.62	0.573
BES cognitive empathy	31	35.29 (4.64)	26	33.92 (3.97)	-1.18	-3.68 to 0.95	0.243
BES affective empathy	31	33.48 (9.22)	26	37.57 (7.37)	1.17	-0.53 to 8.05	0.245

In bold are marked the p values <0.05. WISC, Wechsler Intelligence Scale for Children; YSR, Youth Self Report; ADHD, attention deficit hyperactivity disorder; RFQu, Reflective Functioning Questionnaire, uncertainty about mental states subscale; YPI, Youth Psychopathic Traits Inventory; CU, callousness-unemotional traits; BES, Basic Empathy Scale.

To explore the associations between the FC in the network identified by NBS and the psychological measures, we conducted a series of analyses. First, we conducted bivariate correlations among the mean connectivity in the sub-network identified using NBS and the psychological measures within each group of adolescents and across the whole sample (IA and CA combined). For the IA group, the number of offences and the time since incarceration were also included in the correlation analysis. The threshold of the *p* values was corrected for multiple comparisons (for the t-tests the value was 0.05/12 = 0.004 and for the correlations analysis 0.05/40 = 0.001). To avoid being overly conservative, we discuss the results both robustly (the results surviving multiple comparison correction) and nominally (*p*<0.05).

Next, we conducted a general linear regression model (GLM), with mean connectivity as dependent variable, group membership as fixed factor, and the behavioral variables as covariates. Group membership was added as fixed factor in the GLM to explore the associations between the psychological variables and the mean connectivity, while accounting for the incarceration status. The variables that were identified as significantly different between the groups, as well as the variables that were significantly correlated with the mean connectivity in each group and across the whole sample were included as covariates in the GLM. Age was also included as covariate in the GLM analysis being previously identified to play a role in resting state FC during adolescence (54, 55). Pseudo R^2^ was computed using the formula: 1 − Deviance/Null Deviance.

#### 2.5.1 Follow-up analysis

Next, we conducted follow-up correlation analyses to better understand the association between FC of brain regions connected in the subnetwork and the psychological characteristics associated with the mean connectivity. More precisely, correlation analyses were run among the behavioral variables that significantly predicted the mean connectivity in the GLM analysis and the FC between each pair of brain regions connected in the subnetwork identified by NBS. Given the exploratory nature of the analysis, a threshold of 0.05 was used for the p value.

#### 2.5.2 Sensitivity analysis

To check if the effects remained after the inclusion of all the psychological variables, a second GLM sensitivity analysis was run, with mean connectivity as dependent variable, group membership as fixed factor and all the psychological variables as covariates. Data analysis was conducted in SPSS, version 26 (https://www.ibm.com/products/spss-statistics).

## 3 Results

### 3.1 Group characteristics - psychological measures

Compared to the CA group, IA showed significantly lower scores in verbal abilities, higher levels of externalizing behaviors, and higher levels of impulsivity, and nominally higher scores in the RFQu (see **Table 1**).

### 3.2 Network-based statistics: group differences in functional connectivity

**Figure 2:**
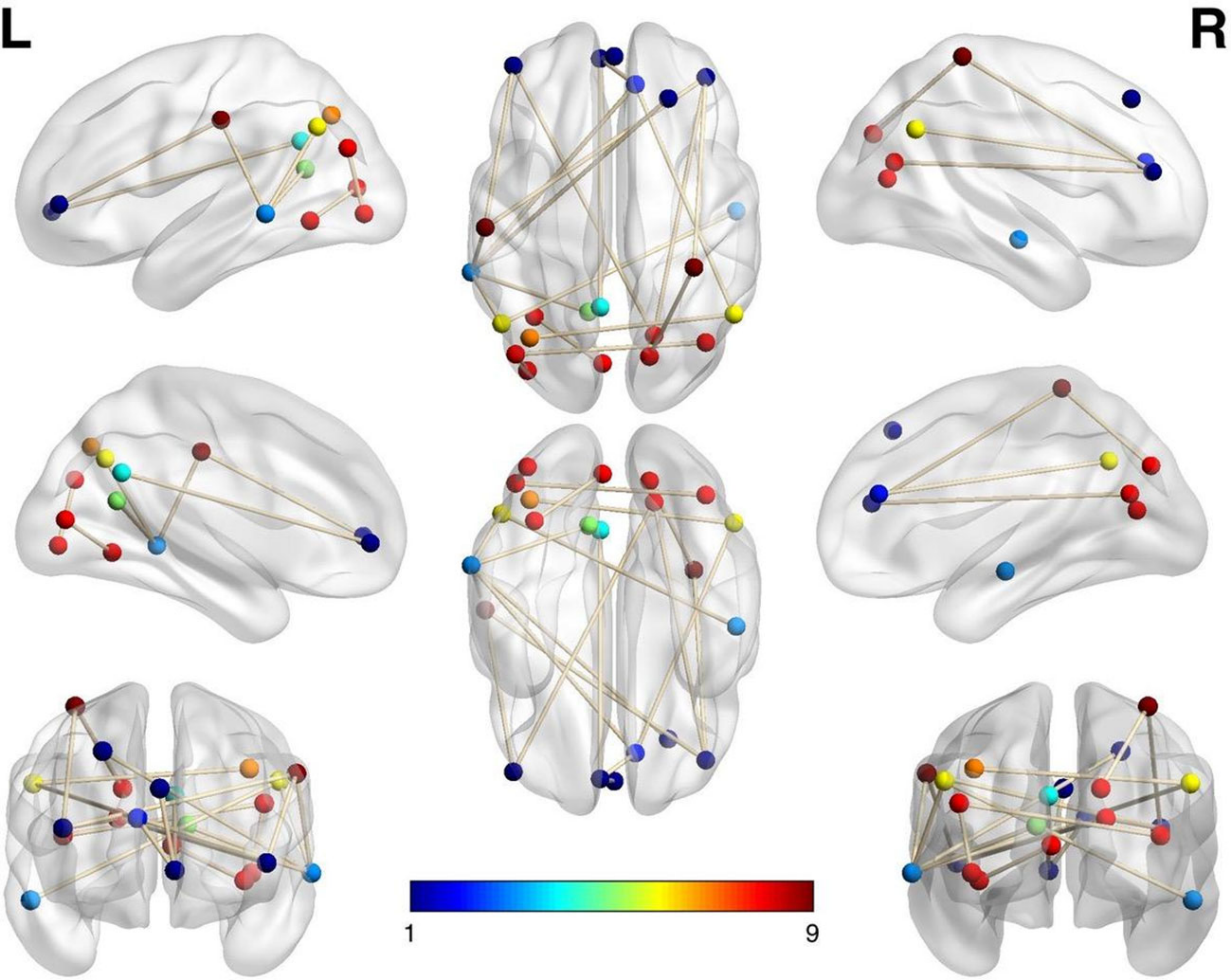
NBS-Subnetwork of increased resting state functional connectivity in the incarcerated adolescents (vs community adolescents). Colored dots represent the stereotactic centroids of brain regions (nodes) defined by Dosenbach functional atlas, and white lines represent suprathreshold links (t >3.5), comprising the affected network identified with the network-based statistic (NBS) (p<0.05. FWE-corrected). Axial views illustrate the involvement of interhemispheric connections (connections between right and left hemispheres). Sagittal views show major involvement of the occipital and frontal lobe in the affected network, medial views show hyper connectivity of DMN-related regions. The arbitrary decided colors only represent the anatomical groups of regions (e.g. Dark Blue nodes are located in the prefrontal area, Red nodes in the occipital).

The NBS analysis identified a single subnetwork showing significant (p<0.05, FWE-corrected for multiple comparisons) increased connectivity in IA (mean connectivity =0.30, SD=0.10) when compared to the CA (mean connectivity = 0.13, SD=0.07). **Figure 2** presents the subnetwork (figure visualized using BRainNEt viewer http://www.nitrc.org/projects/bnv/), comprising 15 edges and 16 nodes (brain regions). This subnetwork predominantly comprised increased connectivity between the occipital and prefrontal region (ventral and dorsal PFC) and prefrontal to prefrontal regions, as well as between anterior cingulate cortex (ACC) and respectively, medial prefrontal cortex and temporal regions. Increased connectivity was additionally found between thalamus node and PFC and between posterior cingulate and temporal regions, as well as between occipital region and basal ganglia. **Table 2** presents all the connections identified as being significantly different between the groups. The subnetwork is located bilaterally with an almost equal number of connections in both hemispheres. The nodes presenting the greatest number of hyper connections are the vmPFC and the occipital nodes. We categorized the Dosenbach regions into corresponding resting state networks based on the classification provided in the article of Dosenbach (51). After comparing the nodes’ coordinates of the subnetwork with those of the Atlas, we could identify nodes belonging to the following well-known resting state networks: 1) *Visual network* with the occipital regions; 2) *Default mode network* with the posterior cingulate, mPFC, and inferior temporal cortex; 3) *Salience network* with dACC and 4) *Frontal network* with the prefrontal regions.

**Table 2:** The values of t-tests for each connection between the nodes that were selected (threshold >3.5) in the subnetwork exhibiting differences between the two groups.

vmPFC to vPFC	3.53
Supfrontal to vPFC	3.77
vmPFC to dACC	3.69
Inftemporal to dACC	3.60
vmPFC to thalamus	3.57
vmPFC to temporal	3.85
postCingulate to temporal	3.53
ACC to temporal	3.97
vPFC to suppParietal	3.90
vPFC to occipital	3.88
vlPFC to occipital	4.05
dlPFC to occipital	4.54
dACC to occipital	3.90
Basal ganglia to occipital	3.59
vFC to occipital	3.58

vmPFC stands for ventromedial prefrontal cortex, ACC for anterior cingulate cortex, dlPFC for dorsolateral prefrontal cortex, d for dorsal, v for ventral, Inf for inferior, Post for posterior.

### 3.3 Associations between resting state functional connectivity and psychological measures

**Table 3** presents the results of bivariate correlations in each group as well as across the whole sample (IA and CA combined) among the mean connectivity in the subnetwork identified by NBS and all psychological measures. In the IA group, mean connectivity was nominally positively correlated with the score of RFQu and nominally negatively correlated with age; in the CA group, the mean connectivity was nominally negatively correlated with interpersonal subscales of YPI. Across the whole sample, the mean connectivity was robustly positively correlated with RFQu, externalizing behaviors, and impulsivity subscale of YPI. **Supplementary Tables S1**, **S2** in the **Supplementary Material** presents the results of bivariate correlation analysis among all the variables in the study.

**Table 3:** Bivariate correlations among the mean connectivity of the subnetwork identified by NBS and the behavioral variables, in each group and across the whole sample.

	Mean connectivity					
	Incarcerated adolescents		Community adolescents		Whole sample	
	*r*	*p*	*r*	*p*	*r*	*p*
Age	-0.39	**0.029**	0.33	0.072	-0.07	0.562
WISC Vocabulary	0.14	0.452	0.22	0.253	-0.19	0.149
BES affective empathy	-0.11	0.549	0.06	0.755	-0.13	0.323
BES cognitive empathy	-0.02	0.892	0.37	0.059	0.19	0.150
RFQu	0.47	**0.008**	0.28	0.163	0.50	**<0.001**
YSR externalizing	0.22	0.224	-0.08	0.660	0.46	**<0.001**
YSR internalizing	0.09	0.630	0.30	0.104	0.20	0.109
YSR ADHD	0.11	0.536	-0.22	0.235	0.13	0.293
YSR thought problems	0.15	0.416	-0.04	0.832	0.16	0.204
YPI CU	0.10	0.578	-0.26	0.176	0.09	0.468
YPI interpersonal	0.18	0.348	-0.51	**0.006**	-0.10	0.431
YPI impulsivity	0.34	0.055	0.01	0.948	0.48	**<0.001**
Number of offences	0.17	0.355	–	–	–	
Time since incarceration	0.29	0.11	–	–	–	

In bold are marked the values of *p <*0.05.

WISC, Wechsler Intelligence Scale for Children; YSR, Youth Self Report; ADHD, attention deficit hyperactivity disorder; RFQu, Reflective Functioning Questionnaire, uncertainty about mental states subscale; YPI, Youth Psychopathic Traits Inventory; CU, callousness-unemotional traits; BES, Basic Empathy Scale.

**Table 4** presents the results of the GLM analysis with mean connectivity as dependent variable, group as fixed factor and the psychological variables that were identified in the previous analyses as different between the groups or nominally and robustly correlated with the mean connectivity (impulsivity and interpersonal dimensions of psychopathy, RFQu, externalizing behaviors, vocabulary scores) as covariates. The GLM showed a significant overall model as suggested by the omnibus test for the mean connectivity (*χ^2^ =* 48.64, *df*=7, *p*<0.001, pseudo *R^2^ =* 0.58). Regarding the association between the mean connectivity and thebehavioral variables, after controlling for the significant effect of group membership, the parameter estimates of the model indicated a significant positive association for RFQu. **Supplementary Figure S1** from the **Supplementary Material** presents the association between the mean connectivity and the scores of RFQu across the whole sample of adolescents.

**Table 4:** The results of the GLM, with the mean connectivity of the subnetwork identified by NBS as dependent variable, group memberships as fixed factor, and behavioral variables as covariates.

Variables	*B*	SE	Wald *χ2*	*p*	95% *CI*
Mean connectivity *(χ ^2^ =* 48.64, *df*=7, *p*<0.001, pseudo *R^2^* =0.58)					
Group (IA as reference)	0.14	0.03	20.71	**<0.001**	0.08 to 0.21
Age	-0.001	0.009	.033	0.958	-0.02 to 0.019
WISC Vocabulary	0.004	0.003	1.10	0.293	-0.003 to 0.01
RFQu	0.014	0.004	10.85	**0.001**	0.006 to 0.02
YSR externalizing	<0.001	0.001	0.01	0.896	-0.02 to 0.01
YPI impulsivity	0.004	0.007	0.35	0.553	-0.01 to 0.01
YPI interpersonal	-0.006	0.005	1.46	0.226	-0.21 to -0.08

In bold are marked the values of *p <*0.05.

WISC, Wechsler Intelligence Scale for Children; CA, community adolescents; RFQu, Reflective Functioning Questionnaire, uncertainty about mental states subscale; YSR, Youth Self Report; YPI, Youth Psychopathic Traits Inventory.

#### 3.3.1 Follow-up correlation analysis

Follow-up correlation analyses were run between the RFQu scores and the FC between each pair of brain regions from the subnetwork identified by NBS, separately in the IA and CA groups and in the whole sample. **Supplementary Table S3** from the **Supplementary Material** shows these results. In the whole sample, we observed positive associations among the RFQu scores and the FC between: i) the occipital lobe to the vPFC, vlPFC, dlPFC, dACC, basal ganglia, vFC; ii) the temporal region to ACC and vmPFC; iii) vmPFC to dACC. In the IA group, the results indicated positive associations among RFQu and the FC between vmPFC to vPFC, supfrontal to vPFC, dACC to occipital, vFC to occipital. In the CA group, the results suggested a positive significant association between RFQu and the FC between dACC to occipital region.

### 3.3.2 Sensitivity analysis

**Supplementary Table S3** from the **Supplementary Material** presents the results. The strength of the association of RFQu remained significant (*B*=0.011, *χ2 =* 7.62, *p*=0.006) and the internalizing problems were significantly associated with the mean connectivity (*B*=0.004, *χ2 =* 7.76, *p*=0.005).

## 4 Discussion

This study represents the first investigation of whole-brain resting state functional connectivity (FC) in a group of incarcerated adolescents (IA) compared to a group of community adolescents (CA) and the correlates with psychopathic traits together with socio-cognitive processes (empathy and reflective functioning RF) and psychological problems. Compared to the CA, IA showed altered FC in a distributed subnetwork including several cortical regions; temporo-parietal regions and mPFC (as part of default mode network- DMN), dACC (as a major node of the salience network -SN), occipital cortex (part of the visual network), and the frontal network, but also subcortical regions, such as basal ganglia and thalamus. We hypothesized that callousness would be associated with reduced resting-state FC, while impulsivity and interpersonal dimensions would correlate with increased FC. These claims were supported by the data; in the IA group, the mean connectivity in the subnetwork was positively correlated with lower reflective functioning levels (e.g. higher uncertainty about mental states-RFQu), whereas in the CA it was negatively correlated with the interpersonal dimension of psychopathy. These results were further supported by correlations in the whole sample of adolescents: the mean connectivity was positively correlated with impulsivity, externalizing behaviors, and RFQu. Finally, linear regression models revealed that the mean connectivity was predicting higher scores on the RFQu, while considering the incarceration status and all the psychological variables.

Our results are in line with previous literature that suggested atypical resting state FC in IA compared to CA (25–27), but also with previous studies on adult and adolescent delinquents (30, 56, 57) and other externalizing behaviors in adolescent and adult populations (58, 59). Using the whole brain NBS analysis, the results of our study add evidence to existing research, suggesting that atypical FC in IA extends to several brain regions, including part of the SN (e.g. dACC), DMN (e.g. parietal, temporal cortex, vmPFC), frontal network (e.g. supfrontal), visual network (e.g. occipital cortex), and subcortical regions (e.g. basal ganglia, thalamus). These brain regions have been previously associated with impairments in different psychological processes that also characterize IA profiles and other populations presenting behavioral problems (58, 60–63), and encompass impairments in self and other processing (vmPFC, mPFC), aberrant reward processing (basal ganglia), lack of impulse control and emotion regulation (dACC, basal ganglia), lack of moral reasoning (mPFC), and weaker integration of interoceptive signals (thalamus and parietal regions). While our findings indicate that these distributed brain regions are involved, it is important to note that the observed relationships between FC alterations and behavioral/psychological impairments are correlational and do not imply direct causality. The NBS approach provides unique insights into the neural correlates of delinquent behaviors by identifying subnetworks that are not easily detected with other methods. For example, the increased connectivity observed in this study between regions of the DMN (e.g., medial prefrontal cortex, posterior cingulate cortex), SN (e.g., dorsal anterior cingulate cortex), and visual and frontal networks suggests widespread alterations in resting-state functional architecture. These findings align with prior research linking DMN dysfunction to impaired self-reflection and moral reasoning in antisocial individuals (61) and SN alterations to deficits in emotion regulation and impulse control (60). By identifying these interconnected subnetworks, NBS highlights how distributed connectivity changes may contribute to core deficits, such as low RF and externalizing behaviors, that characterize delinquent adolescents. Furthermore, the distributed subnetwork identified in this study suggests potential compensatory mechanisms, where increased connectivity in some regions might reflect attempts to offset deficits in others. This is consistent with studies showing that adolescents with high impulsivity may exhibit hyperconnectivity in frontal regions to compensate for reduced top-down control (29). Unlike traditional methods, NBS captures these complex, distributed patterns, offering a more comprehensive understanding of the neural mechanisms underlying delinquent behaviors.

The observed distributed regions affected in the subnetwork suggest that the heterogeneity of IA profiles might only be captured by whole brain analyses. While it is possible for highly focal effects to exist in one given network, most networks - being interconnected, by definition, are likely to show secondary consequences of any focal effects that can propagate along interconnected pathways. Therefore, most effects are likely to influence interconnected subnetworks that the NBS is well suited to detect. In the present study, it seems that IA adolescents present a differential resting state architecture involving regions from several “independent” networks. The distinct interactions between subregions of the networks are correlated with behavioral and psychological impairments observable in IA, more specifically reduced RF, impulsivity and externalizing behaviors. Additionally, these associations might reflect compensatory mechanisms aimed at enhancing the cognitive, motor, and emotional control, despite high levels of impulsivity at the behavioral level (64). Thus, previous studies investigating only a subnetwork might only look at its association with individual variables characterizing IA impairments. However, we acknowledge that the alterations in the observed subnetwork do not necessarily sustain or cause impairments in reflexive functioning (RF). Rather, they may reflect a complex interplay of factors, including neurodevelopmental trajectories and environmental influences, that contribute to these socio-cognitive processes. We could also consider the hypothesis of delayed maturation in the delinquent group, particularly given that we observed a negative correlation between age and mean connectivity. The maturational lag has been proposed in the literature on antisocial behaviors and psychopathy (65), and some studies have linked maturational delays in brain development, such as slow-wave activity to antisocial traits (66). Further, brain development during adolescence is influenced by different developmental factors, such as childhood trauma, prenatal factors, and environmental factors (67). Interestingly, early alcohol abuse has been shown to impair the maturation of brain regions responsible for cognitive control, such as the prefrontal cortex, which is crucial for regulating emotional impulses (68). This disruption can result in long-lasting effects on executive functioning, impulse control, and decision-making, potentially increasing susceptibility to antisocial behavior in later adolescence and early adulthood (69).

The association with RF impairments is not surprising, as the identified altered subnetwork includes regions evidenced to play a role in mental state reasoning, encompassing cortical midline structures, medial PFC, anterior cingulate cortex, and medial posterior parietal cortices (70–72), and the temporoparietal junction (73, 74). Mentalizing, especially the usage of reflective functioning in the context of attachment, has previously been reported as mediating the association between psychopathic traits and proactive aggression in a community sample of typically developing adolescents (75, 76). In our study, the association between low RF and FC in extended brain regions related to different theoretical frameworks, suggests that social-cognitive impairments in delinquent populations might be associated with atypical whole brain connectivity and integration, not limited to the regions previously associated with social cognition, such as the DMN (61). This result comes in the continuity of previous studies suggesting that RF and executive functions, such as impulse control are distinct, but interdependent processes (77). However, it remains unclear which neural mechanisms are shared, and which ones are specific to each of these higher-order processes. Future research should further explore these associations to better understand these mechanisms.

Our results might provide new insights on the mechanisms and neural basis potentially linked to low RF, not only in the IA, but also in CA. Increased resting state FC identified in our study included brain regions previously associated with self-reflection, self-other differentiation, and inference of others’ beliefs, which might explain the self-centered, egocentric bias and the impairments in perspective taking previously observed in IA (39, 78). Additionally, increased connectivity between occipital cortex and different brain regions from SN (dACC) and frontal regions might indicate that IA rely to a greater extent on sensorial, external stimuli, in the detriment of internal, abstract cognitions, which might lead to a bottom-up social cognitive style (79) and ultimately resulting in an external orientated, concrete thinking about mental states and their impact on behaviors (15, 76, 80–82). Further, we could explore the possibility that reduced RF may reflect a broader developmental lag, influenced by both biological and environmental factors. Biologically, this lag could be linked to maturation of brain networks involved in social cognition, such as PFC and temporo-parietal junction, essential for mentalizing and understanding others’ emotions and perspectives. These areas gradually mature throughout adolescence and early adulthood, suggesting that impairments in RF may result in part from delayed cerebral maturation (83). Environmentally, factors such as neglect, early adverse events, or insufficient social interactions during critical developmental periods can further disrupt the maturation of these brain networks. Limited opportunities for reciprocal friendships, group affiliation and social learning might also inhibit the capacity to reflect on mental states, leading to reduced RF (84). Thus, a combination of biological maturation delays and reduced social interactions may contribute to deficits in RF, particularly in populations presenting high levels of antisocial behavior and/or emotional dysregulations. Nevertheless, these interpretations need confirmation from future studies focusing on the neural basis of RF and their role in the development of delinquent behaviors during adolescence. In addition, most previous studies used task-based functional MRI to assess the association between FC and behavioral or socio-cognitive impairments related to IA. Contrasting with previous studies observing a decrease in FC in IA (25, 26) and in community adolescents presenting antisocial behaviors (25, 36, 59, 85–87), our results indicate that, compared to the CA, the IA presented only increased resting state FC. Notably, previous studies showed that the impulsivity dimension of psychopathy was associated with increased resting state FC (29, 88–92), whereas the emotional callousness dimension was associated with decreased resting state FC in adolescents and adults presenting antisocial behaviors (30, 58, 93), which could explain these discrepancies. This result might be explained by the characteristics of our subgroups. IA adolescents presented significantly higher impulsivity and externalizing behaviors and lower verbal IQ and RF than the CA. However, the two subgroups did not differ in the emotional callousness, interpersonal dimensions, empathy scores or any other psychopathology problems scores. These group characteristics might suggest that IA presented mainly reactive, impulsive forms of delinquent behavior. Furthermore, the CA subgroup might also be a heterogenous group in terms of psychopathology profiles, since the two subgroups do not differ in the ADHD scores, which is consistent with previous studies suggesting that ADHD symptoms is also prevalent in the general population, especially in boys (94). This is further sustained by our findings, suggesting that in the whole sample (IA and CA combined), higher levels of impulsivity and externalizing behaviors were positively correlated with the increase in connectivity in the subnetwork identified by the NBS. These results might suggest that the subnetwork identified by the NBS is not specific to delinquency, but connects regions involved in emotional and behavior dysregulations, present in both normative and clinical populations presenting externalizing behaviors. Future studies should replicate our findings using the NBS approach in IA groups presenting high levels of CU traits.

In summary, while the interpretation of case-control differences in resting-state functional MRI data may not always be straightforward (95), the NBS identified a core network of increased FC that is consistent with psychopathological alterations reported in the literature. Furthermore, findings of the present study could be explained as a cascade of associated processes. Our results suggest that the increased connectivity in the subnetwork identified by the NBS might be associated with processes involved on one hand in low socio-cognitive abilities, namely low RF, and in behavioral manifestations such as impulsivity and externalizing behaviors. We could hypothesize that, the increased FC observable in IA, driven by impairments in RF associated with a bottom-up cognitive style, will translate to externally oriented, concrete thinking about mental states, which in turn could lead to impairments in self-regulation manifesting as impulsivity and externalizing behaviors. Contemporary developments in mentalization-based therapy are currently evaluating the value of focusing on such processes in psychological therapy for adolescents with conduct disorder (96, 97), a youth group sharing some characteristics with the IA group in this study. Future studies should also address individual factors, such as early adverse events, which may further contribute to the understanding of RF impairments in juvenile offenders (15).

## 5 Limitations

There are limitations to this study that need to be acknowledged. First, some of the brain regions identified as part of the subnetwork are relatively large, with substructures that have been previously related to different functions (e.g. basal ganglia). Further, while the identified subnetwork encompasses widely distributed regions, it reflects a significant and relevant pattern of altered connectivity that warrants further investigation. Future studies might use alternative connectivity methods following hypothesis-driven analyses, or more targeted network analyses to refine the understanding of these connectivity changes. Secondly, the cross-sectional design limits our developmental interpretation; future longitudinal studies conducted on IA are needed to confirm the implication of RF and the neural mechanisms at stake in the emergence of antisocial behaviors. We acknowledge that while the increased connectivity observed in the subnetwork correlates with behavioral and socio-cognitive measures, the nature of these relationships requires further exploration. Future longitudinal studies, ideally incorporating both resting-state and task-based fMRI paradigms, could help elucidate the causal pathways and mechanisms underlying these associations. Additionally, social-cognitive processes and psychopathic traits were investigated using self-reported questionnaires, which represent screeners requiring further in-depth exploration of these psychological processes. Future studies need to focus on these variables using other types of measures, such as experimental tasks or interviews. Furthermore, the sample size of the study is relatively small, and the results must be replicated on larger samples. The sample was also composed only of males, it would be interesting to investigate if the results can be replicated in female samples. Finally, although the number of days since admission to the juvenile detention center and the MRI testing was not significantly correlated with the resting state connectivity in the subnetwork identified by NBS, other factors associated with incarceration (e.g. social isolation, stress etc.) that we did not consider in the present study could influence the NBS results. All the variables entered in the GLM model (including group membership) explained 58% of the variance in the resting state connectivity, thus other variables (e.g. low socio-economic status, low IQ, childhood trauma, alcohol and drug use) might explain the resting state connectivity differences. Future studies must continue investigating the psychological variables associated with whole brain resting state connectivity in IA, ideally using matched samples on these possible confounding variables. Of note, the present study aimed to investigate the differences in resting state FC between two groups of IA and CA, and their correlations with psychological factors, it is therefore limited to these objectives and cannot be generalized to index the risk for delinquent behaviors or to qualify as biomarkers of delinquency.

## 6 Conclusion

Overall, our study extends previous research by identifying a unique subnetwork of brain regions that presented increased resting state FC in a group of IA, compared to CA. The increased connectivity in this subnetwork was uniquely associated with socio-cognitive impairments, namely low reflective functioning. While providing understanding of whole brain resting state FC in IA, our results might also provide some insights about the impaired mechanisms implicated in the socio-cognitive processes during adolescence.

## Data Availability

The raw data supporting the conclusions of this article will be made available by the authors, without undue reservation.
