# Supplementary material for: Atypical functional connectome is associated with low reflective functioning in incarcerated adolescents
Source: Front Psychiatry. 2025 Jan 10;15:1385782. doi: 10.3389/fpsyt.2024.1385782 (PMC11757290; doi:10.3389/fpsyt.2024.1385782)
Supplement: Supplementary file 1 [file DataSheet1.docx]

**Supplementary material**

**Atypical functional connectome is associated with low reflective functioning in incarcerated adolescents**

Derome Mélodie^1,2*^& Morosan Larisa^1,2*^, Eliez Stephan^2^, Heller Patrick^3,4^, Debbané Martin^1,2,5^

**Contents**

[Table S1. Bivariate correlations among the mean connectivity in the subnetwork identified by the NBS and all the variables in the incarcerated adolescents group (IA) below and community adolescents (CA) above the table. 2](#_Toc103348304)

[Table S2. Bivariate correlation analysis among the mean connectivity of the subnetwork identified by the NBS, using the whole sample of adolescents (IA and CA combined). 3](#_Toc103348305)

[Figure S1. Correlation between the RFQ uncertainty about mental states subscale and the mean connectivity in the subnetwork identified by the NBS analysis across the whole sample of adolescents. 4](#_Toc103348306)

[Table S3. Results of the follow-up correlation analysis between the functional connectivity between each pair of nodes from the subnetwork and the RFQu scores in the whole sample and within each group separately. 5](#_Toc103348307)

[Table S4. The results of the GLM sensitivity analysis, with the mean connectivity of the subnetwork identified by the NBS as dependent variable, group memberships as fixed factor, and behavioural variables as covariates. 6](#_Toc103348308)

# **Table S1**. Bivariate correlations among the mean connectivity in the subnetwork identified by NBS and all the variables in the incarcerated adolescents group (IA) belowthe diagonal and community adolescents (CA) above the diagonal.

| **Variables** | **1** | **2** | **3** | **4** | **5** | **6** | **7** | **8** | **9** | **10** | **11** | **12** | **13** | **14** | **15** |
| --- | --- | --- | --- | --- | --- | --- | --- | --- | --- | --- | --- | --- | --- | --- | --- |
| 1. Mean connectivity |  | .33 | .22 | .06 | .37 | .28 | -.08 | .30 | -.22 | -.04 | -.26 | -.51** | .01 | - | - |
| 2. Age | -.39 * |  | -.01 | .15 | .20 | .32 | .11 | .11 | -.04 | .19 | .01 | -.15 | -.13 | - | - |
| 3. WISC Vocabulary | .14 | -.49** |  | .12 | .06 | .05 | -.11 | .06 | -.13 | .15 | -.13 | -.24 | .18 | - | - |
| 4. BES affective | -.11 | .07 | .007 |  | .38 | .11 | -.04 | .49* | .19 | .42* | -.48* | -.16 | .10 | - | - |
| 5. BES cognitive | -.02 | -.15 | .12 | .33 |  | .05 | -.25 | .33 | -.25 | -.03 | -.22 | -.25 | .02 | - | - |
| 6.RFQu | .47** | -.11 | -.11 | -.05 | .04 |  | .18 | .28 | -.10 | .30 | .07 | .06 | .02 | - | - |
| 7. YSR externalizing | .22 | -.20 | -.07 | -.09 | -.09 | .24 |  | .18 | .42* | .19 | .18 | .20 | .49** | - | - |
| 8. YSR internalizing | .09 | .10 | -.37* | .33 | -.20 | .27 | .34 |  | -10 | .59** | -.34 | -.21 | .10 | - | - |
| 9. YSR ADHD | .11 | -.18 | -.17 | -.13 | -.18 | .12 | .46** | .24 |  | .19 | .15 | .24 | .41* | - | - |
| 10. YSR thought | .15 | -.14 | -.24 | .16 | .01 | .37* | .45* | .66** | .38* |  | -.23 | .07 | .08 | - | - |
| 11. YPI CU | .10 | -.05 | -.03 | -.69** | -.34 | .19 | .21 | -.20 | .12 | .17 |  | .29 | .10 | - | - |
| 12. YPI interpersonal | .18 | -.15 | .15 | -.11 | -.17 | .04 | -.009 | -.001 | .16 | .09 | .33 |  | .27 | - | - |
| 13. YPI impulsivity | .34 | -.32 | .31 | -.24 | -.08 | .25 | .40* | -.28 | .27 | .09 | .49* | .34 |  | - | - |
| 14. Number offences | .17 | -.56** | .52** | -.29 | .15 | .19 | .06 | -.47** | <.001 | -.24 | .10 | .03 | .38* |  |  |
| 15. Duration since incarceration | 0.03 | -.05 | -0.11 | -.30 | -.06 | .18 | -.09 | -.02 | .04 | .11 | .29 | .12 | -.08 | .18 | - |

WISC: Wechsler Intelligence Scale for Children; BES: Basic empathy scales; RFQu: Reflective Functioning Questionnaire uncertainty about mental states subscales; Youth Self Report; ADHD: Attention deficit and hyperactivity disorder manifestations; YPI: Youth Psychopathic Inventory; CU: callous-unemotional traits

*p<.05; **p<.01

| **Variables** | **1** | **2** | **3** | **4** | **5** | **6** | **7** | **8** | **19** | **10** | **11** | **12** | **13** |
| --- | --- | --- | --- | --- | --- | --- | --- | --- | --- | --- | --- | --- | --- |
| 1. Mean connectivity |  |  |  |  |  |  |  |  |  |  |  |  |  |
| 2. Age | -.07 |  |  |  |  |  |  |  |  |  |  |  |  |
| 3. WISC Vocabulary | -.19 | -.23 |  |  |  |  |  |  |  |  |  |  |  |
| 4. BES affective | -.13 | .12 | .12 |  |  |  |  |  |  |  |  |  |  |
| 5. BES cognitive | .19 | -.02 | .02 | .32* |  |  |  |  |  |  |  |  |  |
| 6.RFQu | .50** | -.08 | -.16 | -.07 | .08 |  |  |  |  |  |  |  |  |
| 7. YSR externalizing | .46** | -.03 | -.33** | -.14 | -.03 | .36** |  |  |  |  |  |  |  |
| 8. YSR internalizing | .20 | .10 | -.20 | .37** | .01 | .25 | .28* |  |  |  |  |  |  |
| 9. YSR ADHD | .13 | -.11 | -.23 | -.02 | -.17 | .11 | .47** | .11 |  |  |  |  |  |
| 10. YSR thought | .16 | .03 | -.09 | .26* | .01 | .34** | .34** | .63** | .31 |  |  |  |  |
| 11. YPI CU | .09 | -.03 | -.13 | -.61** | -.27* | .21 | .24 | -.23 | .16 | .02 |  |  |  |
| 12. YPI interpersonal | -.10 | -.15 | .01 | -.13 | -.21 | .03 | .02 | -.09 | .17 | .06 | .30* |  |  |
| 13. YPI impulsivity | 48** | -.19 | -.05 | -.13 | .05 | .29* | .63** | -.02 | .40** | .15 | .34** | .21 |  |

# **Table S2**. Bivariate correlation analysis among the mean connectivity of the subnetwork identified by the NBS, using the whole sample of adolescents (IA and CA combined).

WISC: Wechsler Intelligence Scale for Children ; BES: Basic empathy scales; RFQu: Reflective Functioning Questionnaire uncertainty about mental states subscales; YSR : Youth Self Report; ADHD: Attention deficit and hyperactivity disorder manifestations; YPI: Youth Psychopathic Inventory; CU: callous-unemotional traits

*p<.05; **p<.01

# **Figure S1.** Correlation between the RFQ uncertainty about mental states subscale and the mean connectivity in the subnetwork identified by the NBS analysis across the whole sample of adolescents.


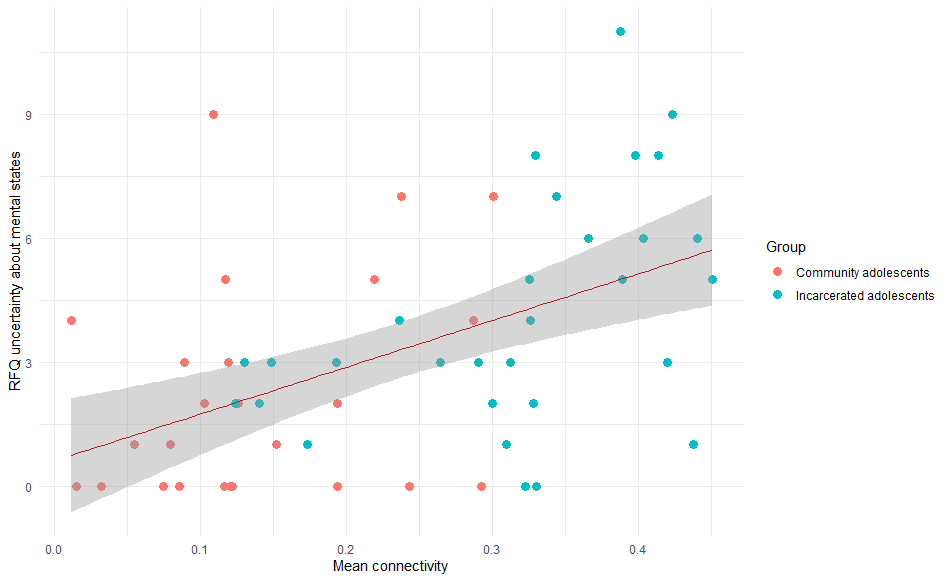


|  | RFQu | | | | | |
| --- | --- | --- | --- | --- | --- | --- |
|  | **Incarcerated adolescents** | | **Community adolescents** | | **Whole sample** | |
|  | ***r*** | ***p*** | ***r*** | ***p*** | ***r*** | ***p*** |
| vmPFC to vPFC | 0.37 | **0.04** | 0.05 | 0.80 | 0.32 | 0.16 |
| Supfrontal to vPFC | 0.37 | **0.04** | -0.13 | 0.5 | 0.25 | 0.05 |
| vmPFC to dACC | 0.21 | 0.26 | 0.13 | 0.51 | 0.29 | **0.02** |
| Inftemporal to dACC | 0.21 | 0.25 | -0.11 | 0.59 | 0.23 | 0.09 |
| vmPFC to thalamus | 0.009 | 0.96 | 0.21 | 0.31 | 0.21 | 0.12 |
| vmPFC to temporal | 0.35 | 0.05 | 0.08 | 0.68 | 0.36 | **0.007** |
| postCingulate to temporal | 0.05 | 0.77 | 0.22 | 0.29 | 0.23 | 0.08 |
| ACC to temporal | 0.20 | 0.27 | 0.06 | 0.75 | 0.29 | **0.03** |
| vPFC to suppParietal | 0.27 | 0.13 | 0.15 | 0.46 | 0.35 | **0.008** |
| vPFC to occipital | 0.20 | 0.28 | 0.19 | 0.35 | 0.32 | **0.01** |
| vlPFC to occipital | 0.25 | 0.17 | 0.34 | 0.08 | 0.40 | **0.002** |
| dlPFC to occipital | 0.30 | 0.1 | 0.15 | 0.44 | 0.38 | **0.004** |
| dACC to occipital | 0.41 | **0.02** | 0.44 | **0.02** | 0.50 | **<0.001** |
| Basal ganglia to occipital | 0.29 | 0.11 | 0.37 | 0.06 | 0.40 | **0.002** |
| vFC to occipital | 0.40 | **0.02** | 0.05 | 0.81 | 0.36 | **0.006** |

**Table S3**. Results of the follow-up correlation analysis between the functional connectivity between each pair of nodes from the subnetwork and the RFQu scores in the whole sample and within each group separately. In bold are marked the p values <0.05.

**Table S4.** The results of the GLM sensitivity analysis, with the mean connectivity of the subnetwork identified by the NBS as dependent variable, group memberships as fixed factor, and behavioural variables as covariates. In bold are marked the values of *p* <0.05

| **Variables** | ***B*** | **SE** | **Wald *χ2*** | ***p*** | **95% *CI*** |
| --- | --- | --- | --- | --- | --- |
| **Mean connectivity *( χ^2^*=56.80, *df*=14, *p*<0.001, pseudo *R^2^* =0.66)** | | | | |  |
| Group (IA as reference) | 0.14 | 0.03 | 19.04 | **<0.001** | 0.78 to 0.20 |
| Age | -0.002 | 0.009 | 0.02 | 0.872 | -0.20 to 0.01 |
| WISC vocabulary | 0.005 | 0.003 | 1.73 | 0.188 | -0.002 to 0.01 |
| YSR externalizing | <0.001 | 0.001 | 0.003 | 0.958 | -0.003 to 0.003 |
| YSR internalizing | 0.004 | 0.001 | 7.76 | **0.005** | 0.001 to 0.007 |
| YSR ADHD | <0.001 | 0.001 | 0.02 | 0.872 | -0.003 to 0.004 |
| YSR thought problems | -0.003 | 0.001 | 2.88 | 0.089 | -0.006 to 0.001 |
| RFQu | 0.01 | 0.004 | 7.62 | **0.006** | 0.003 to 0.02 |
| YPI CU | -0.005 | 0.007 | 0.54 | 0.461 | -0.02 to 0.009 |
| YPI impulsivity | 0.005 | 0.007 | 0.55 | 0.456 | -0.009 to 0.02 |
| YPI interpersonal | -0.002 | 0.004 | 0.16 | 0.685 | -0.01 to 0.008 |
| BES cognitive empathy | 0.005 | 0.002 | 2.76 | 0.096 | -0.001 to 0.01 |
| BES affective empathy | -0.002 | 0.001 | 1 | 0.315 | -0.006 to 0.002 |

WISC: Wechsler Intelligence Scale for Children; YSR: Youth Self Report; ADHD: attention deficit hyperactivity disorder; RFQu: Reflective Functioning Questionnaire, uncertainty about mental states subscale; YPI: Youth Psychopathic Traits Inventory; CU: callousness-unemotional traits; BES: Basic Empathy Scale
